# Cerebellar Infarction in Childhood: Delayed-Onset Complication of Mild Head Trauma

**Published:** 2016

**Authors:** Ibrahim Ilker OZ, Evrim BOZAY OZ, Ismail ŞERIFOĞLU, Nurullah KAYA, Oktay ERDEM

**Affiliations:** 1 Department of Radiology, BulentEcevit University, School of Medicine, Zonguldak, Turkey; 2 Department of Anesthesiology and Reanimation, Zonguldak State Hospital, Zonguldak, Turkey; 3 Department of Radiology, Medipol Hospital, Istanbul, Turkey

**Keywords:** Brain infarction, Trauma, Vasospasm, MR imaging

## Abstract

**Objective**

Cerebellar ischemic infarction is a rare complication of minor head trauma. Vertebral artery dissection, vasospasm or systemic hypo perfusion can cause infarct. However, underlying causes of the ischemic infarct cannot be explained in nearly half of cases. The accurate diagnosis is essential to ensure appropriate treatment. Here we report a five yr old boy patient of cerebellar infraction after minor head trauma, admitted to emergency serves of BulentEcevit University, Turkey in 2013. We aimed to remind minor head trauma that causes cerebellar infarction during childhood, and to review the important points of the diagnosis, which should be keep in mind.

## Introduction

Cerebellar infarction is rare in children especially after minor head injury (MHI). A patient with MIH has a small but important risk of traumatic brain injury that requires early identification. Traumatic brain injury depending on accelerationdeceleration forces is commonly seen as diffuse axonal injury, contusion and epidural or subdural hematoma (1). Furthermore ischemic brain infarction can be seen after traumatic brain injury, and it is usually located in the cerebral hemispheres. Here we report a five yr old boy with isolated left superior cerebellar artery territory infarction after MIH. The clinical and radiological features of cerebellar infarction in children with MIH were delineated and the literature was reviewed.

## Case Report

**Fig 1:**
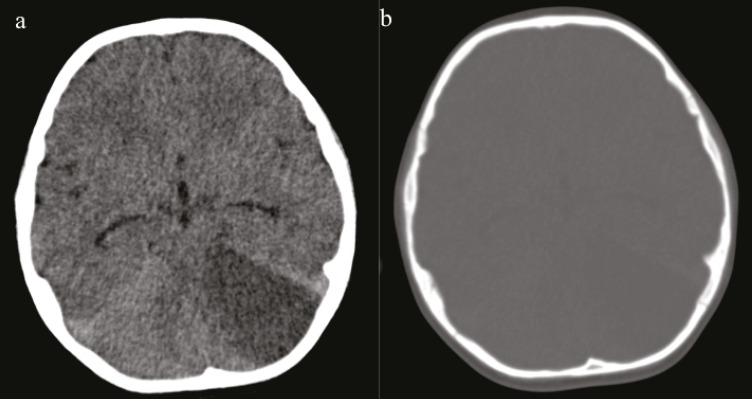
Non-contrast CT shows hypodens area with mass effect due to edema in the left cerebellar hemisphere (a). At the same level, there is no fracture of bone structure (b).

**Fig 2:**
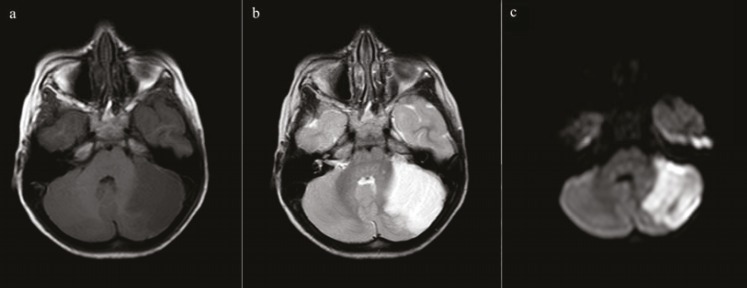
MRI and DWI show an infarction at the superior cerebellar artery territory in the left cerebellar hemisphere. The Infarction is seen as hypointensity on T1 weighted images (a), hyperintensity on T2 weighted images (b), hyperintensity suitable for restricted diffusion on DW images

**Fig 3:**
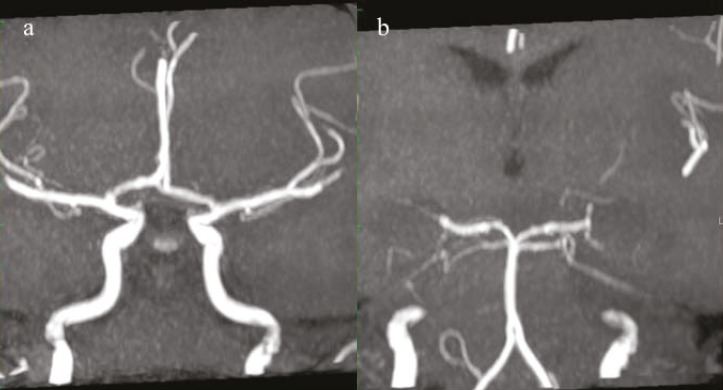
MR angiography shows normal arterial tree on both anterior circulation (a) and posterior circulation (b

**Fig 4:**
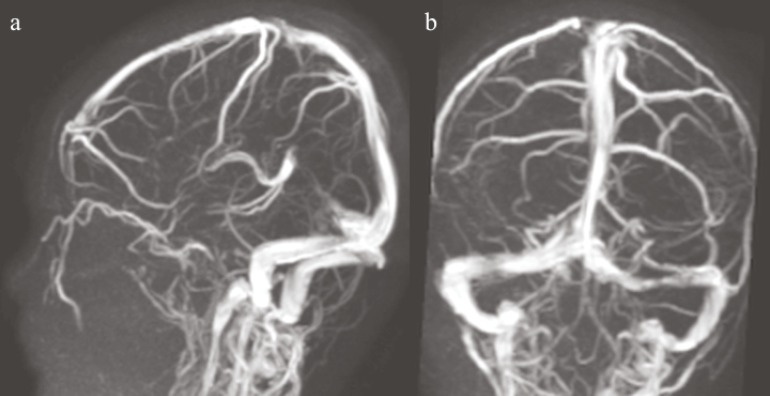
MR venography shows normal venous drainage (a) with signal loss in the left transverse sinus because of anatomical variation (b

A five yr old boy patient was admitted to emergency service of BulentEcevit University, Turkey; due to balance disorder and headache in April 2013. He said that his head hit the stove while playing a game at home a day ago. There was no loss of consciousness and seizure disorder. Twenty four hour later, vague balance disorder developed. At the time of admission, his vital signs, physical exam, and laboratory results were completely normal. Gait was minimally ataxic. Cerebellar system, sense examination and other neurological exams were normal. On computed tomography (CT) scan, a wide hypodens area was seen in the left cerebellar hemisphere (Figure 1a). There was no evidence of fracture and intra or extra parenchymal hemorrhage (Figure 1a-b). Magnetic resonance imaging (MRI)was performed to reveal infarction. MRI and diffusion weighted imaging (DWI) showed an acute infarction with significant edema on the territory of the left superior cerebellar artery (SCA) (Figure 2 a-c). On MR, angiography there was no vascular abnormality (Figure 3). Signal loss was visualized in the left transverse sinus on MR venography. It was evaluated as a variation in the size of the left transverse sinus (Figure 4). Doppler ultrasonography of carotid-vertebral arteries and echocardiogram were normal. Analysis of factor V Leiden, prothrombin G20210A and MTHFR C677T gene mutations was made for prothrombotic genetic predisposition. Any genetic mutation was not detected. After exclusion of cardiac, genetic risk factors and dissection of vertebral artery, vasospasm was accepted as the etiology of cerebellar ischemia. The patient’s parents provided written informed consent to participate in the study.

## Discussion

Infarction after MHI is often seen in middle cerebral artery territory which affects basal ganglia (2). Posttraumatic cerebellar infarction is very rare and a superior cerebellar artery territory infarction is less common than the posterior inferior cerebellar artery, although the superior cerebellar artery is the largest branch of the posterior circulation (3).Dissections, endothelial intimal injuries, or vasospasms are potential reasons of post-traumatic infarction (4). During head trauma, an opposing movement of the brain parenchyma produces stretching and shearing effects on the vessels. This causes vasospasm, endothelial intimal injury, or dissection. Most of the cases presented in medical literature are related with cerebral perforating arteries (4). Intermittent arterial spasm is the most common cause of brain infarctions after trauma (2). However, arterial dissections cause 20% of ischemic infarcts in children and young adults thus dissection should be eliminated in patients with traumatic infarction (5). Neurological deficits of post-traumatic infarction were reported at 15 min to 72 h after trauma (6). In addition, in traumatic pediatric patients, typical time to onset of vasospasm appears to peak at days 2 to 3, and duration of vasospasm is 2–4 d (7). The time between onset of symptoms and trauma is an important evidence for vasospasm. CT scan is the most commonly used emergent modality for stroke, and is widely available, acquires images quickly, and accurately eliminates acute hemorrhage (8). In the first hours after acute infarction, CT scan is usually negative. CT scan has lower sensitivity in the posterior fossa, because of the bone artifacts of the skull base. MRI is more superior to CT scan showingearly acute infract changes, and MRI with DWI is more sensitive than conventional MRI (8, 9). The clinician should communicate any clinical concern for stroke especially related to trauma to the radiologist so that DWI sequences are acquired. When cerebellar infarction is diagnosed, the next step is angiography (catheter, CT angiography, MR angiography) to reveal underlying vascular pathology. The advantages of CT angiography are easily accessible and short imaging time. However, ionizing radiation especially in children is the most important disadvantage. The requirement of contrast agent is another disadvantage. Though noncontrast MR angiography eludes the handicaps of CT angiography, more time is necessary to acquire images compared with CT scan. In addition, MR venography may be performed at the same time and helpful to diagnosis of venous thromboembolism. Catheter angiography is the gold standard (8). It is invasive and has all disadvantage of CT angiography. Highly skilled personnel for angiography are needed. It should be preferred for interventional treatment. In conclusion, vasospasm after minor head injury also causes delay onset of symptoms. To better outcomes, detailed questioning of trauma and detailed investigations for vasospasm should be included in the evaluation of cerebellar infarctions.

## Author Contribution

Ibrahım Ilker Oz: Drafting, designing of the work, and final approval of the work. 

EvrimBozay Oz: Drafting, designing of the work, and final approval of the work. 

Ismail Şerifoğlu: Interpretation, and final approval of the work. 

Nurullah Kaya: Interpretation, and final approval of the work. 

OktayErdem: Final approval of the work. 

All authors agreement to be accountable for all aspects and integrity of the work.

## Conflict of interest

The authors declare that they have no Conflict of interest.
